# Recycling of Mechanically Ground Wind Turbine Blades as Filler in Geopolymer Composite

**DOI:** 10.3390/ma14216539

**Published:** 2021-10-30

**Authors:** Kinga Pławecka, Jakub Przybyła, Kinga Korniejenko, Wei-Ting Lin, An Cheng, Michał Łach

**Affiliations:** 1 Chair of Materials Engineering, Faculty of Material Engineering and Physics, Cracow University of Technology, Jana Pawła II 37, 31-864 Cracow, Poland; jakubprzybyla55@gmail.com (J.P.); kkorniejenko@pk.edu.pl (K.K.); michal.lach@pk.edu.pl (M.Ł.); 2 Department of Civil Engineering, National Ilan University, No. 1, Sec. 1, Shennong Rd., Yilan City 260, Taiwan; wtlin@niu.edu.tw (W.-T.L.); ancheng@niu.edu.tw (A.C.)

**Keywords:** geopolymer, wind turbine blades, recycling

## Abstract

This paper concerns the recycling of waste material from wind turbine blades. The aim of the research was to determine the possibility of using ground waste material derived from the exploited structures of wind turbines as a filler in geopolymer composites. In order to determine the potential of such a solution, tests were carried out on three different fractions originating from the ground blades of wind turbines, including an analysis of the morphology and chemical composition of particles using SEM and an EDS detector, the analysis of organic and inorganic matter content and tests for multivariate geopolymer composites with the addition of waste material. The compression and flexural strength, density and absorbability tests, among others, were carried out. The composite material made of the geopolymer matrix contained the filler at the level of 5%, 15% and 30% of dry mass. The addition of the filler showed a tendency to decrease the properties of the obtained geopolymer composite. However, it was possible to obtain materials that did not significantly differ in properties from the re-reference sample for the filler content of 5% and 15% of dry mass. As a result of the research, it was found that waste materials from the utilization of used wind power plants can become fillers in geopolymer composites. It was also found that it is possible to increase the strength of the obtained material by lowering the porosity.

## 1. Introduction

Renewable energy sources in the form of wind farms are an important part of the strategy in the fight against global warming. Unfortunately, this energy is not entirely free of environmental impacts, and one of the significant problems is the disposal/management of wind turbine blade waste [1]. The European Composites Industry Associaton (EuCIA) has estimated that the mass of composite materials used to manufacture wind turbines is already 2.5 million tonnes. In 2050, the mass of these materials is expected to reach 43 million tonnes, where 25% will be in Europe, 40% in China, 16% in the United States and 19% in the rest of the world [2].

Composite materials used for wind turbine blades are mainly polymers (resins) reinforced with various fibers. Owing to the use of such reinforcements, it is possible to obtain the desired properties; e.g., the addition of carbon and glass fibers increases the strength of the composite material while maintaining a relatively low weight of the entire element. Another example of material solutions used for wind turbines is natural composites [3,4,5,6,7]. The manufacturing industry of wind turbines largely uses ter-modified polymers. Unfortunately, after the life cycle of a wind blade is over, there is a problem with the disposal of the material [8].

Currently, 85% to 90% of the mass of a wind turbine can be recycled. Methods are known for the disposal of the foundations, tower and nacelle components [9]. Excluding the foundations, 6% of the wind turbine mass is rubber, plastic and fiber-reinforced and other composites, which are mainly found in the rotor blades [8]. The overall composition of the rotor blade is as follows: it contains approximately 93% composite material, 2% PVC, 2% balsa wood and 3% metals, paints and putty [10].

Currently, there are processes for recovering material from turbine blades, which can be divided into mechanical, thermal and chemical methods. Mechanical grinding is based on grinding the material into small particles using mills or other equipment. This method is relatively low cost and requires a low energy input [11]. The crushed material obtained can be used as a filler or reinforcement or for cement production. The disadvantage of this process is that the properties of the recovered fibers are lowered and that long fibers cannot be obtained [12].

A windmill propeller contains a large amount of organic matter in its material composition. Storing this type of waste in the ground is considered to be dangerous for the environment. Cherrington et al. compared the disposal options for waste from wind turbines in their study. The study shows that the best alternative for the processing of used wind turbines would be the invention of a material system with the possibility of recycling in a closed loop [13]. A solution could also be the use of more natural materials: mainly natural fiber reinforcements, such as flax, hemp, jute and bamboo fibers.

Researchers have developed the possibility of creating wind turbine blades with the addition of flax fiber. In comparison with other natural fibers, and when combined with a polymer matrix, they have better mechanical properties [14,15]. Unfortunately, due to the high content of cellulose in flax fibers as a result of the brittle fracture of the material, delamination occurs, which is a serious design problem in wind turbine blades [4]. Additionally, this material (flax fiber) is characterized by a high moisture absorption, so, in order to minimize this process, it is necessary to treat the fibers. The most common method is sorting, which improves the surface wettability and thus contributes to a more efficient load transfer between the polymer matrix and the flax fibers (reduction of delamination) [5].

Girisha K.G. et al. compared the feasibility of using different polymer matrices (polyester and epoxy) with hemp and jute fibers. The experiment proved that the polyester matrix is a better solution because of the obtained test results: higher values of the bending strength, tensile strength and impact strength [16]. Research carried out on hybrid composites with the addition of natural fibers indicates that they can be used as a material of low mass in situations where there is a low load on the element. In order to increase the strength of the polymer composite reinforced with natural fibers, further processing is necessary, which would increase the adhesion forces between the matrix and fibers [17]. 

Hardhik Bhanushali et al. developed a woven glass fiber composite with carbon nanotubes between the intermediate layers. The polymer–nanotube (CNT) composite exhibited very good mechanical properties during testing compared to traditional materials used for wind turbine blades [18].

For the conducted research on the possibility of recycling composite materials originating from windmill turbines, a geopolymer material was selected as the matrix due to its unique features. It is estimated that the synthesis of geopolymers consumes two to three times less energy and emits four to eight times less CO_2_ than the production of Portland cement. Studies have shown that geopolymer concrete has a high compressive strength, good acid resistance, low shrinkage and better binding of heavy metals compared to Portland-cement-based concrete.

A life cycle assessment is an analytical tool to measure the impact of the environmental performance of products over their life span, from extraction and operation to recycling or final disposal [19]. At present, several LCA studies on the environmental impact of cement [20] or concrete [21,22] have been developed by researchers. The geopolymer material as an alternative to conventional concrete shows great potential to improve the environmental performance [23]. The main environmental charges in the LCA cycle from geopolymers are related to the use of alkane activators during their production. An alternative to the above action is the possibility of using natural zeolites as raw materials used in the synthesis of geopolymer materials [24,25].

Geopolymers are a group of modern construction materials with a range of properties that allow them to replace common engineering materials, such as traditional concretes and building composites. Geopolymers have many advantages over traditional concretes, of which, the most important are [26,27,28,29,30,31,32,33,34,35]:

High compressive and bending strength (depending on the recipe and hydrothermal treatment, compressive strength from 15 MPa to 100 MPa, flexural strength from 3 MPa to 25 MPa),

- A very high acid resistance and resistance to chlorides and sulphate;
- A resistance to weather conditions, including a very high freeze resistance;
- A high heat resistance (they do not lose their strength properties at temperatures up to 800 °C, and, in fact, their strength may increase. The decrease in strength is observed only at temperatures from 800 to 1000 °C);
- A low porosity (similar to natural granite). The porosity can be controlled by changing the ratio of the amorphous phase to the crystalline phase in the geopolymer, so geopolymers with a very low porosity (below 2.5%) and geopolymers with a high porosity (above 20%) can be obtained;
- No corrosion of steel reinforcement in the geopolymer;
- A high adhesion of the geopolymer to steel;
- A faster onset of setting time compared to concretes;
- Dimensionally stable (no or little shrinkage on setting).

Additionally, geopolymers can be produced from various types of waste materials [36,37]. Such a material may be a prospective material to be used as a reinforcement or a simple filler of waste materials from wind power plants withdrawn from use. It is a particularly interesting material because of the fibers present, which can function as reinforcement in geopolymer composites [38,39].

One of the most commonly used raw materials for geopolymers is fly ash. The composition of fly ash varies greatly, depending on the origin, type of coal burned, parameters and combustion technology. It usually contains between 90% and 99% inorganic matter, between 1% and 9% organic matter from unburned coal and less than 0.5% liquids. Inorganic matter consists of 34% to 80% amorphous phase and 17% to 63% crystalline phase, and contains mainly SiO_2_, Al_2_O_3_, Fe_2_O_3_ and CaO. In geopolymer composites and concretes, fillers and aggregates are also used as in conventional concretes. In order to reduce the consumption of natural aggregates and sand, and, at the same time, solve the problem of waste from wind turbine blades, it is possible to use waste fillers in various types of concretes and composites. This paper describes the results of testing geopolymer composites with the addition of fillers (replacing part of the sand and also having the role of reinforcement) in the form of recycled waste from wind turbine blades [40,41,42,43,44,45].

The creation of geopolymer composites reinforced with waste fibers may contribute to a wider use of geopolymers in housing and municipal construction. The end-of-life wind turbine blade waste additive is introduced as a mixture of shredded fibers and resin (matrix) and can serve two roles: fibers as a dispersed reinforcement and resin as a filler to replace other fillers in construction materials, such as sand. It would be a very good material for various types of facade cladding in the form of tiles, material for construction of sidewalks, walkways, garden accessories, etc. The geopolymer matrix provides a very good resistance to corrosive environments, and waste fibers will contribute to the reduction of brittle fracture, with which geopolymers currently have a problem.

All of the advances, innovative approaches and ideas regarding the use of waste materials in the production of concrete and geopolymer composites are becoming more important and valuable. This is due to several factors, including many countries tightening policies on material reuse, reducing the use of natural resources and reducing CO_2_ emissions. It is also related to the problem of the availability of materials used so far to produce geopolymers, such as fly ash and slag. It should be noted that these raw materials are no longer as available as they were in the 1980s, 1990s and early 2000s. Many steel mills are closing in Europe and production has moved to Asia. This situation is similar with coal combustion and fly ash.

Current policies in many countries are moving away from coal combustion, which results in less fly ash being available. At the moment, even cement plants that use fly ash have problems with supplying this raw material. Although some deposits from many years ago are still available, they will soon run out. Even if some countries continue to generate fly ash from coal combustion for a few decades, the price of fly ash will be so high that it will no longer be economically attractive to use it in geopolymerization processes. These prices already reach EUR 15-30 per Mg in Europe, and only a few years ago, they could be purchased at the price of transport costs. Another problem raised by the realisation of the research described in this article is the utilisation of problematic waste from the recycling of used wind turbine elements. This problem will grow in many parts of the world. The issue of the possibility of using waste from decommissioned wind turbines in geopolymers, described in the paper, is innovative and not commonly presented before.

The implementation of this type of technology would allow for a number of benefits in the future, the most important of which are:

- The utilization of problamatic waste ground fiber resins;
- A reduction of CO_2_ emissions;
- A reduction in the consumption of fillers in concrete (sand, etc.).

## 2. Materials and Methods

### 2.1. Materials and Samples Preparation

**Figure 1:**
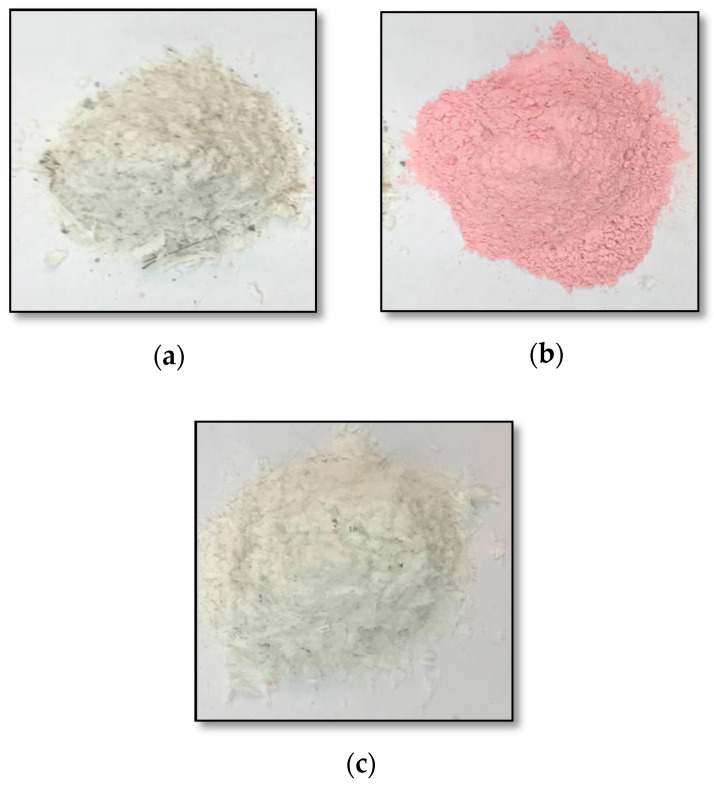
Wind turbine waste fractions from: (**a**) the aerodynamic part of the rotor blade—fraction I; (**b**) monolithic part of the turbine blade, which is used to fasten the entire blade element—fraction II; (**c**) the aerodynamic part of the rotor blade—fraction III.

In this study, an attempt was made to produce geopolymers using three different waste fractions from wind turbine rotors (Geopolymer Building Eko, Żagań, Poland). Conventional class F fly ash was obtained from the Skawina Heat and Power Plant (Skawina, Poland) and river sand (ZODIAK, Świętochłowice, Poland). Figure 1 shows a subset of the different waste fractions: materials I, II and III. The waste fraction I shown in Figure 1a is white-gray in color. It contains visible fibers and powder particles. The fibers are clumped into elongated pieces and flakes ranging from a few millimeters to a few centimeters in length. In addition, small gray particles are present, which are usually between 0.5 mm and 3 mm in size. This fraction is derived from the aerodynamic part of the rotor blade. The waste fraction II marked in Figure 1b is pink in color. It is in the form of a fine powder with no distinct fibers in its composition. This fraction is derived from the monolithic part of the turbine blade, which is used to fix the whole blade component. The waste fraction III marked in Figure 1c is white in color. It contains fibers and powder particles. The fibers are joined together to form elongated pieces and flakes ranging from a few millimeters to a few centimeters in length. This waste is derived from the aerodynamic part of the rotor blade. Materials I and III differ only in the degree of fragmentation. Material I is smaller, more fragmented and homogeneous. Material 3 shows fragments of laminates (resin-bonded fibers) in the form of tiles, etc.

Materials used for testing are glass fibers (the type was not specified) and epoxy resin matrix. With regard to the materials used for turbine blade structures, the most commonly used reinforcement fiber is E-type glass fiber (borosilicon fiber). The thickness of the glass fibers used is usually in the range from 10 µm to 20 µm. The mass of glass fiber in the composite material can be up to 75%. For the production of relatively long rotor blades, E-glass does not have sufficient properties. Therefore, carbon or glass fiber with a modified composition of type S, S2 (magnesium–aluminum–silicon) or R (calcium–aluminum–silicon) is used. These are used to increase the modulus of elasticity and reduce the weight of the component. Carbon fiber is the most durable, but it also has the highest price. Therefore, hybrid fibers created on the basis of combination of the above-mentioned materials are used [46,47]. However, new materials are constantly being searched for, and cheaper aramid, basalt, cellulose or polyethylene fibers are in demand. The polymer matrix is most often created based on thermosetting resins. Epoxy, polyester or vinylester resins are used. They have low viscosity and can be cured at low temperature. For relatively large rotors, epoxy resins are mostly used. Their main purpose is to bind the fibers together [45,47]. Matrices can also be created from polyurethanes or thermoplastics [48].

The alkaline activator was a solution of 8 M sodium hydroxide and R-145 sodium water glass with a molar modulus of 2.5 and a density of approximately 1.45 g/cm^3^. An aqueous solution of sodium hydroxide of a given concentration was mixed with water glass at a ratio of 1:2.5 by weight. The solution was prepared according to the scheme: technical hydroxide flakes were solubilized in water, and then an aqueous solution of sodium silicate was added. The components were mixed and allowed to reach a constant concentrate. The dispersion of waste in the geopolymer mass was ensured by mixing the components, such as fly ash, sand and waste fractions, dry for 15 min in a rotary mixer until a homogeneous mixture was obtained. Such mixture was then poured with alkaline solution while stirring continuously. In this way an even dispersion of the waste in the geopolymer mass was ensured. The formation of agglomerates or other unfavorable phenomena was not observed. To prepare the mass, the precursors were mixed with the activator for approximately 10 min and poured into molds. The molds were placed on a vibrating table to remove air bubbles. After preparing the masses, the samples were covered with foil and placed in a laboratory dryer (SLW 750 STD, Pol-Eko-Aparatura, Wodzisław Śląski, Poland) for 24 h at 75 °C. The samples were deformed and cured under laboratory conditions (temperature approximately 20 °C, relative humidity approximately 50%) for 28 days. After this time period, strength tests were conducted. Table 1 contains the names of the samples for better systematization and the mixing ratios of the products. Different L/S ratios were used in this study due to the need to obtain identical consistency of the geopolymer mass for the different variants, which was only possible with varying amounts of liquid. Due to the different absorbency of the waste materials, different amounts of alkali activator were used, but this was very meticulously measured and the actual values are given in the table below. Varying the amount of liquid can affect the properties of different composites, but using the same amount of liquid leads to some materials being too thin and others not being able to be formed properly.

### 2.2. Methodology

#### 2.2.1. Analysis of Organic and Inorganic Content

Waste material from windmill turbines was combusted in a chamotte kiln to determine the organic and inorganic fractions. After measuring 80 g of each type of fraction, it was placed in ceramic crucibles and then transferred to the furnace. The material was fired for 12 h at 600 °C. After the firing process, the samples were weighed again and their masses were determined using a RADWAG PS 200/2000.R2 (RADWAG, Radom, Poland) laboratory analytical balance (maximum load: 200/2000 g; reading accuracy: 0.001/0.01 g).

#### 2.2.2. Microscopic Observation

Particular fractions of wind turbine waste materials were observed microscopically in order to determine the structure formation. Additionally, their chemical composition could be determined using EDS. Microscopic observations were carried out using a JEOL JSM-6390LV scanning electron microscope (JEOL Ltd., Tokyo, Japan). Before testing, the surface of the sample was sputtered with a gold layer using a JOEL JEE-4X vacuum sputtering machine (JEOL Ltd., Tokyo, Japan).

#### 2.2.3. Density

The geometric method was used to determine the true density of manufactured geopolymers based on wind turbine waste, fly ash and sand. The density was determined as the average of measurements for 3 samples. The samples were measured with a laboratory caliper with a measurement accuracy of 0.01 mm, and the mass of the samples was determined using a RADWAG PS 200/2000.R2 laboratory analytical balance (maximum load: 200/2000 g; reading accuracy: 0.001/0.01 g).

#### 2.2.4. Strength Tests

Flexural strength tests were carried out according to EN 12390-5 (“Testing of hardened concrete. Flexural strength of specimens”) using a Matest 3000 kN universal testing machine (Matest, Treviolo, Italy). The dimensions of the specimens were 50 mm × 50 mm × 200 mm. The spacing between the support bars was 150 mm. The test speed was set at 0.5 MPa/s. Compressive strength tests were performed according to EN 12390-3 (“Testing of hardened concrete. Compressive strength of specimens”) on cubic specimens (50 mm × 50 mm × 50 mm) using the same Matest 3000 kN testing press at the same speed. The strength tests were conducted after 28 days of specimen conditioning. The number of samples tested in the flexural test was a minimum of 4 for each type of material (approximately 40 in total). In the compression test, 8 specimens from each type of geopolymer were tested (80 in total). The results obtained are average values.

#### 2.2.5. Absorption Testing

Measurements of absorbability of the geopolymer material were conducted in accordance with PN-EN 206+A1:2016-12 standard (“Concrete—Requirements, properties, production and conformity”), on cubes measuring 40 mm × 40 mm × 37-57 mm (height). The samples were placed in plastic dishes on stands and then flooded with water to a level equal to half the height of the samples. After 24 h, the samples were additionally flooded with water to a level approximately 10 mm higher than the height of the samples. They were then weighed after at least 24 h until the weight was stable (no more than 0.2%). The next step was to place the samples in a dryer (SLW 750 STD, Pol-Eko-Aparatura, Wodzisław Śląski, Poland) at 75 °C. After at least 24 h, they were weighed again.

## 3. Results and Discussion

### 3.1. Analysis of Organic and Inorganic Content Results

**Figure 2:**
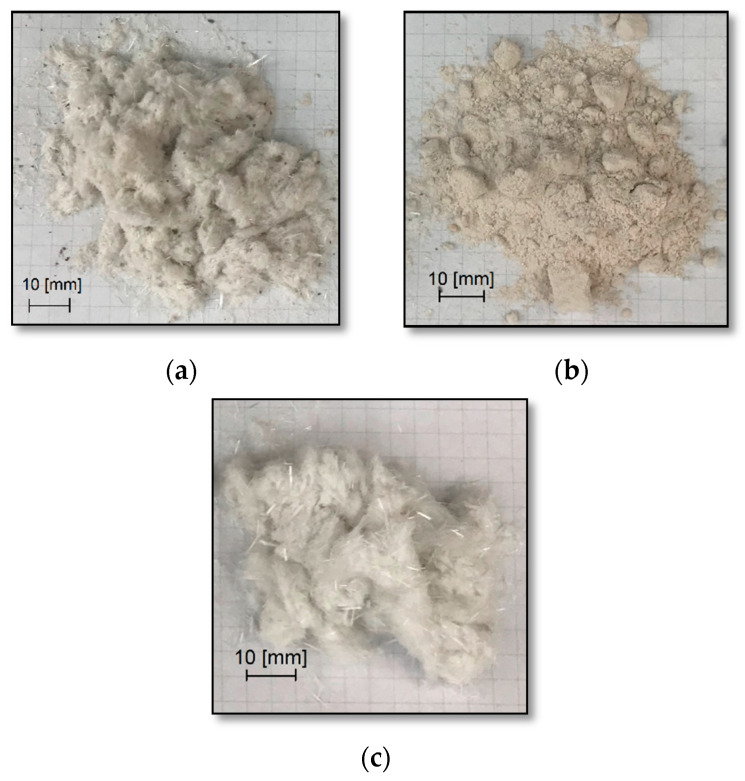
Wind turbine waste fractions subjected to a firing process at 600 °C, for 12 h: (**a**) rotor blade aerodynamic part—fraction I; (**b**) turbine blade monolithic part—fraction II; (**c**) rotor blade aerodynamic part—fraction III.

Figure 2a–c show the fractions of the waste material from the windmill turbines after the completion of the firing process of the organic parts in the chamotte furnace. The burning was carried out at 600 °C for 12 h. These materials changed their color under the influence of temperature. The fibers, which were less visible in the unburned material, also became visible. Table 2 shows a summary of the results obtained during the above test. The inorganic fiber content is highest for fraction I (72.35%) and fraction III (70.88%). Fraction II was characterized by a lower content of the inorganic part, which was 56.89%. This means that it contained the lowest proportion of the reinforcement of all three fractions of the windmill turbine waste. The weight loss of the samples is not only a result of disposing of combustible organic parts, but also due to some effects appearing from moisture (but this is of secondary importance). The samples were stored under the same conditions, and possible mass loss effects due to moisture should be the same for the three tested waste fractions.

### 3.2. Structure Observations and Chemical Composition of Precursors

**Figure 3:**
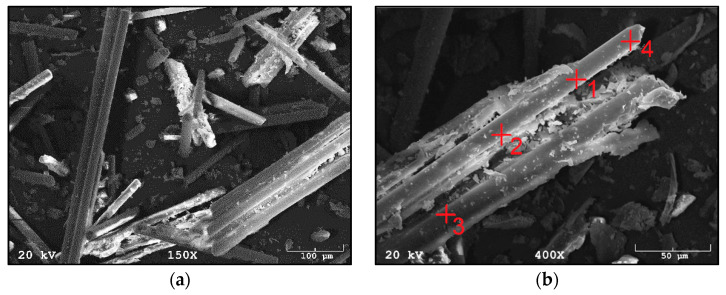
SEM images of fraction I from wind turbines: (**a**) in 150× magnification; (**b**) in 450× magnification.

**Figure 4:**
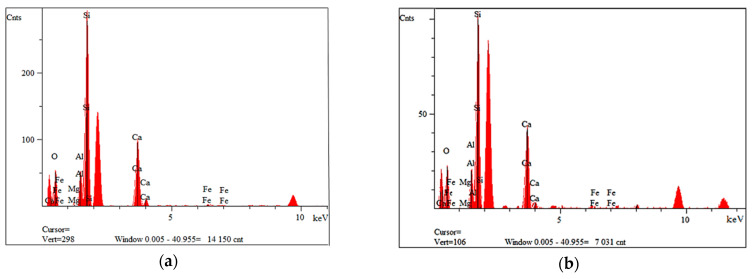
EDS analysis of fraction I from wind turbines: (**a**) for point 2 of Figure 3b; (**b**) for point 3 of Figure 3b.

Figure 3 shows the morphology of waste material number I, a composite additive in the tested geopolymer materials based on fly ash and river sand. The composite was characterized by the presence of fibers whose length oscillated between 50 µm and 1000 µm. The fibers were glued together in small agglomerates or separately. Fine particles could be seen on the surface of the fibers. These are most likely the residue of the polymer matrix. This waste fraction also contained small particles that were less than 50 µm in size, which may have arisen from fiber fragments or crushed parts of the polymer matrix. Figure 4a,b show the measured spectra, recorded with the EDS system, on the basis of which, it was possible to qualitatively determine the chemical composition of the tested material. Fraction I showed the presence of elements such as oxygen, aluminum, magnesium, calcium, silicon and iron.

**Figure 5:**
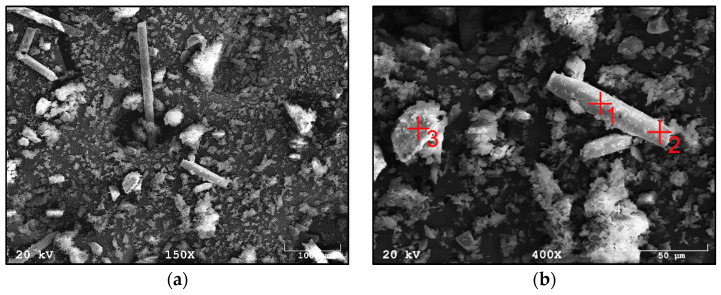
SEM images of fraction II from wind turbines: (**a**) in 150× magnification; (**b**) in 450× magnification.

**Figure 6:**
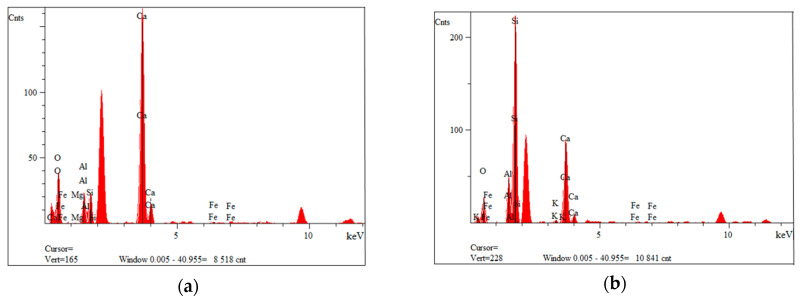
EDS analysis of fraction II from wind turbines: (**a**) for point 1 of Figure 5b; (**b**) for point 2 of Figure 5b.

Figure 5a,b show the waste material fraction II, which is derived from the monolithic part of the windmills. The composite fraction numbered II has the least amount of fibers compared to composite numbers I and III. The fibers do not form aggregates with each other and occur singly. Their length varies from 50 µm to 400 µm, but mainly fibers of a length of around 100 µm can be observed. Remnants of the polymer matrix are present on the surface of the material. Fraction II contains a significant amount of fine, irregular particles, whose size is oscillated below 25 µm. Figure 6a,b show the measured spectra, recorded with the EDS system, on the basis of which, it was possible to qualitatively determine the chemical composition of the tested material. Fraction II showed the presence of elements such as oxygen, sodium, silicon, calcium, aluminum, magnesium and iron.

**Figure 7:**
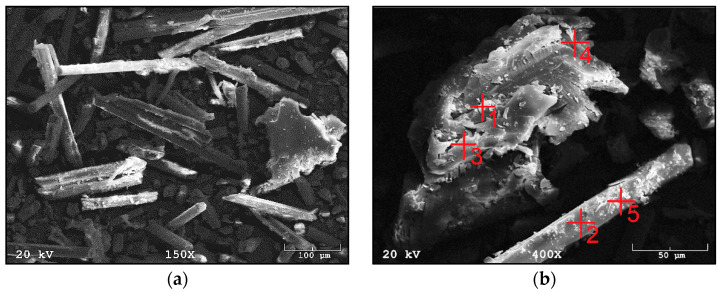
SEM images of fraction III from wind turbines: (**a**) in 150× magnification; (**b**) in 450× magnification.

**Figure 8:**
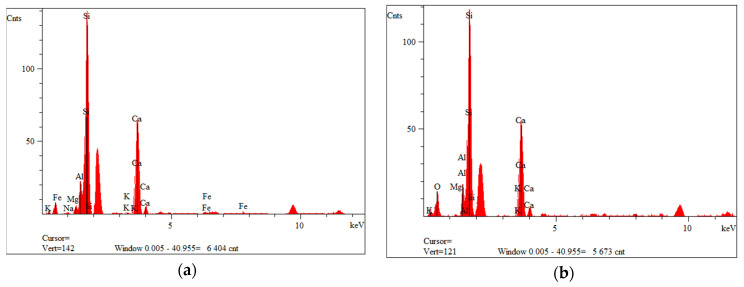
EDS analysis of fraction III from wind turbines: (**a**) for point 3 of Figure 7b; (**b**) for point 5 of Figure 7b.

Figure 7a,b show a fraction of waste material III, which is derived from windmill rotors. The composite is characterized by a large number of fibers, which occur either singly or as agglomerates. The agglomerates concentrate several fibers joined together. Compared to composite I, the agglomerates are larger and stuck together with a large amount of the polymer matrix. Fiber lengths between 100 µm and 1000 µm are mainly visible. The fraction also has particles that are less than 50 µm in size, which may have arisen from fiber fragments or crushed parts of the polymer matrix. Figure 8a,b show the spectra recorded with the EDS system, on the basis of which, it was possible to qualitatively determine the chemical composition of the material studied. Fraction III showed the presence of elements such as oxygen, aluminum, silicon, potassium, calcium and iron.

For all fiber types, elements such as silicon, aluminum and sodium are observed in the chemical composition. This proves that the glass fibers used are type R fibers. Type R fiber is strong and corrosion resistant, and is made from a calcium–aluminum–silicate mixture.

### 3.3. Density Results

**Figure 9:**
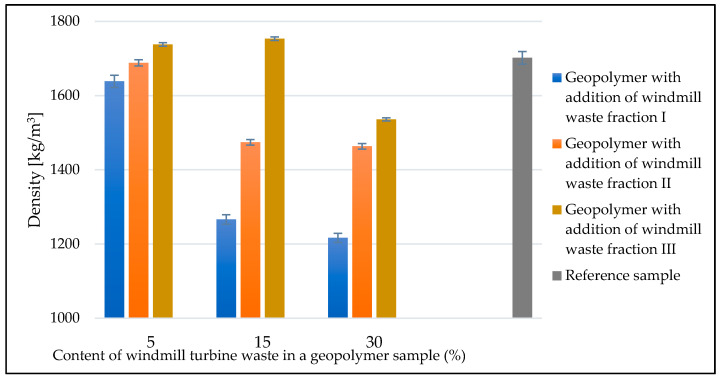
Medium density of geopolymer materials with addition of windmill fractions.

The density of geopolymer materials based on fly ash and sand, with the addition of waste fractions from windmill turbines, was calculated using the geometric method. The average values for each type of geopolymer are shown in Figure 9. The samples with the addition of the waste fraction tended to decrease in density as the proportion of the filler phase increased. In each series, samples with the addition of waste fraction I had the lowest density and samples with the addition of waste fraction III had the highest density. Composite II in each series had a density between waste fraction I and waste fraction III. The largest differences in density were seen for the waste fraction contribution of 15%, where the discrepancy between samples was over 400 kg/m^3^, and for the waste fraction contribution of 30%, where the discrepancy was approximately 300 kg/m^3^. Densities in the vicinity of the reference sample were obtained for composite III with fraction contents of 5% and 15%. The lowest density was obtained with the addition of the 15% and 30% fraction of composite I. It was 1266 kg/m^3^ and 1217 kg/m^3^, respectively, where, in comparison, the reference sample had a density of 1701 kg/m^3^. The lowest increase in density, compared to the reference sample, was observed in the sample with fraction II at a 30% addition to the geopolymer.

The results of the density testing are also related to the density of the waste itself, and the greatest influence here has the matrix of laminates waste (resin), whose density can be as high as 1000 kg/m^3^. This can contribute to a significant reduction in composites involving such a material, and this was observed in the presented study, as the lowest density samples were those with a 30% (the highest) proportion of waste. The density of glass fibers is on the level of 2000–2600 kg/m^3^ and is similar to (or slightly higher than) the geopolymer matrix.

The porosity of the material was the main reason for the density changes in the tested samples. No correlation was observed between the amount of liquid (L/S ratio) and the density of the material.

The differences in the density of the samples are a result of the pores formed, which may be due to several reasons. Macro- and ultramacropores visible in the photographs are formed mainly at the fiber/matrix interface, but they are also a result of an improper compaction of these materials (the aeration of the geopolymer mixture at the time of mixing the components). Although there is no clear correlation between the density of the samples (porosity) and the L/S ratio, it must be remembered that the liquid/solid ratio affects the porosity, so it will also affect the density of the samples. However, this is a very complex relationship, and it also depends on the amount of waste additive introduced and its hydrophilicity.

### 3.4. Mechanical Properties

Strength tests, such as compressive strength, are the basis for assessing the correctness of the geopolymerization process, as well as for evaluating the suitability of the prepared materials. The starting raw materials for geopolymers strongly influence the resulting microstructure, and thus affect the subsequent properties of the finished mortar. The compressive strength of geopolymer materials depends on several factors, such as the structure, the presence of a crystalline phase, the content and strength of the gel phase, the distribution and hardness of insoluble Al-Si particles and the surface reaction between the gel phase and insoluble Al-Si particles [48].

**Figure 10:**
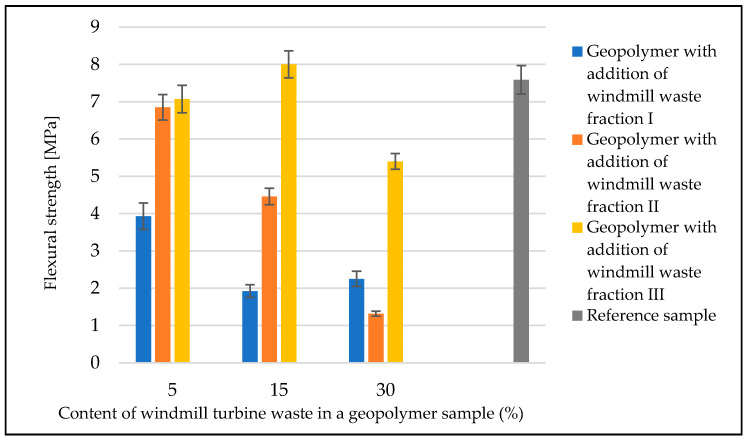
Flexural strength of geopolymer samples with the addition of waste fractions from windmill turbines.

**Figure 11:**
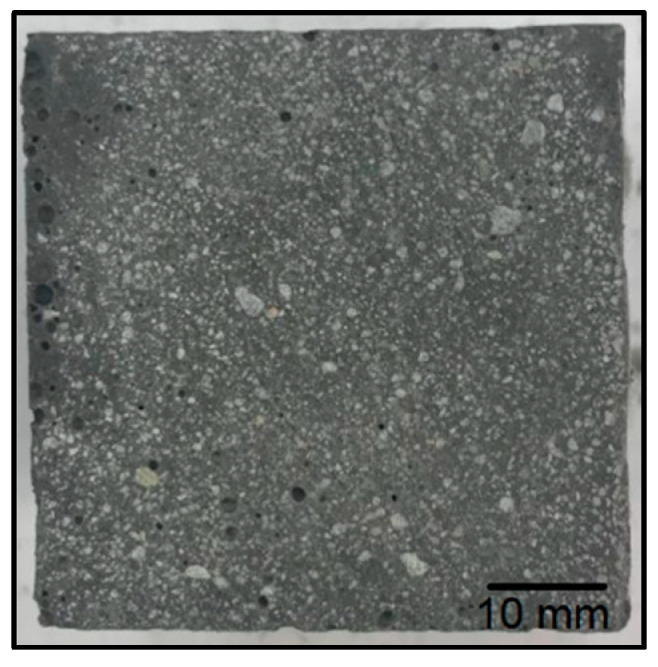
Breakthrough of the reference sample.

**Figure 12:**
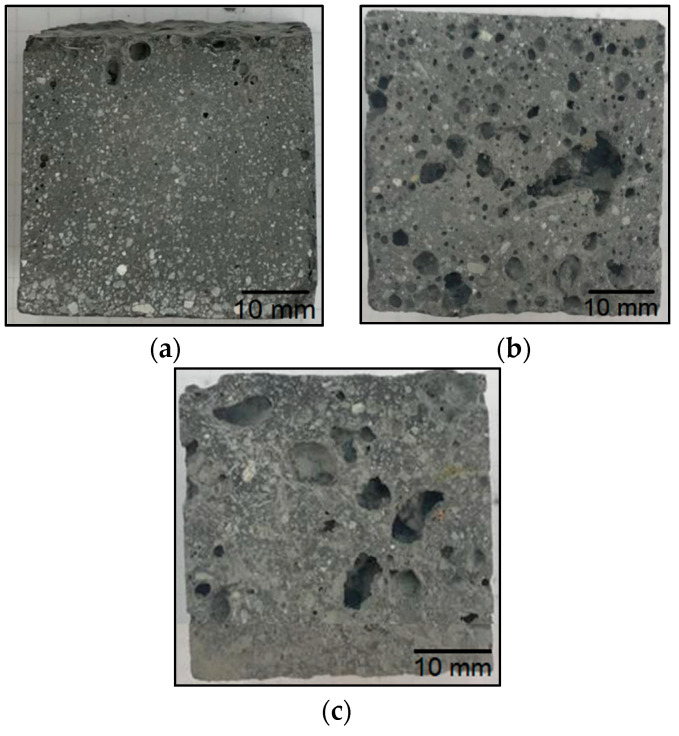
Breakthroughs of geopolymer samples based on fly ash and river sand with the addition of fraction I from a windmill turbine: (**a**) 5% fraction addition; (**b**) 15% fraction addition; (**c**) 30% fraction addition.

Figure 10 shows a summary of the strength tests that were performed for each type of geopolymer. For the reference material (Figure 11), the average flexural strength was 7.6 MPa. The material with the addition of waste fraction I (Figure 12) showed a decrease in flexural strength compared to the reference sample. Depending on the degree of the addition of waste fraction I, the flexural strength values for geopolymers were as follows: 3.9 MPa for 5%, 1.9 for 15% and 2.8 for a 30% fraction addition.

**Figure 13:**
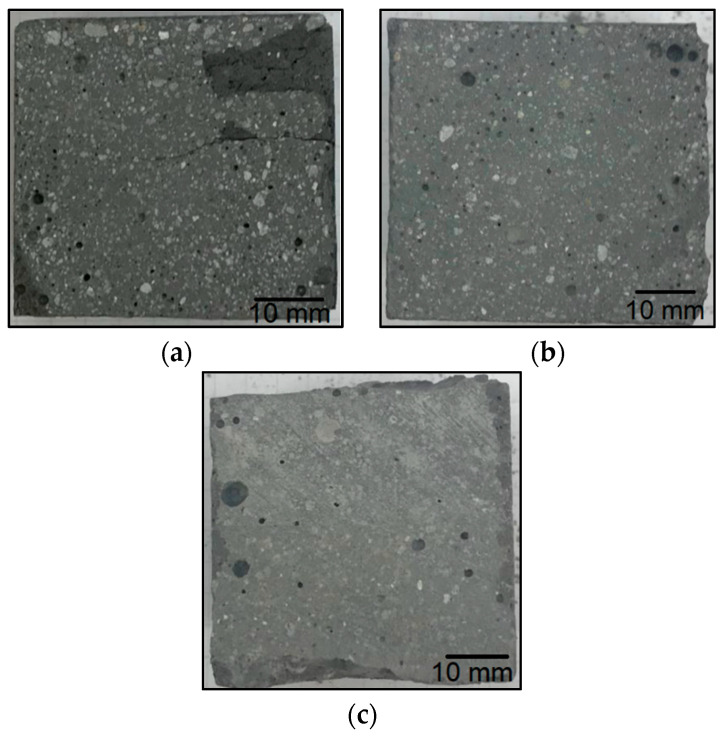
Breakthroughs of geopolymer samples based on fly ash and river sand with the addition of fraction II from a windmill turbine: (**a**) 5% fraction addition; (**b**) 15% fraction addition; (**c**) 30% fraction addition.

The material with the addition of the waste II fraction (Figure 13) showed a decrease in strength with an increase in the proportion of the waste phase. Samples with a 5% waste fraction addition obtained similar result (6.9 MPa) to the reference sample. The flexural strength values for this group of material were: 4.5 MPa for a 15% fraction addition and 1.3 MPa for a 30% fraction addition to the geopolymer mass.

**Figure 14:**
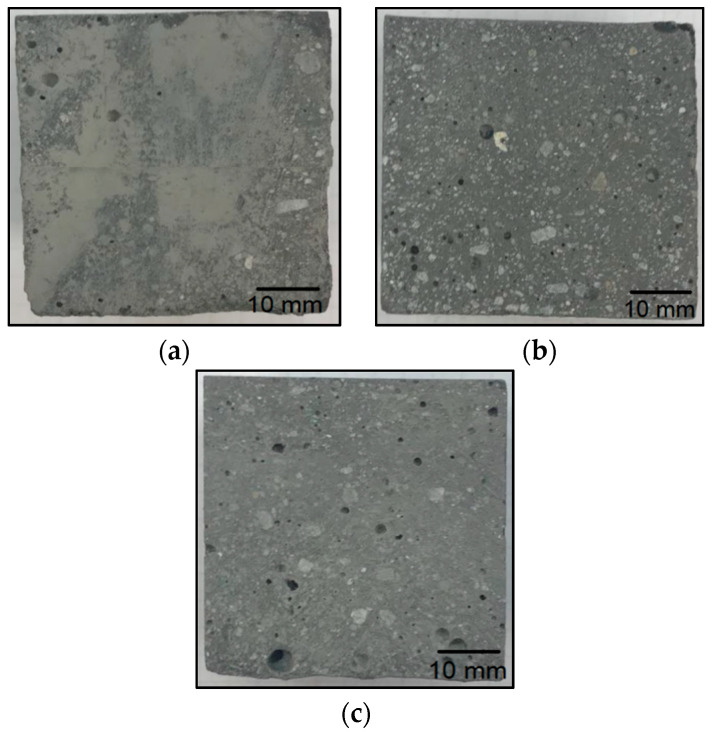
Breakthroughs of geopolymer samples based on fly ash and river sand with the addition of fraction III from a windmill turbine: (**a**) 5% fraction addition; (**b**) 15% fraction addition; (**c**) 30% fraction addition.

The material with waste fraction III (Figure 14) showed the highest flexural strength relative to the materials obtained from waste fractions I and II. The samples with 5% and 30% waste fraction showed a decrease in flexural strength (7.1 MPa and 5.4 MPa, respectively) compared to the reference geopolymer sample. In this series, the addition of fraction III of an amount of 15% contributed to an increase in flexural strength (8.0 MPa) in comparison to the reference material. The decrease in strength properties is mainly related to the decrease in the active cross section of geopolymer due to the introduced additives and also due to increased porosity. The fibers present in the waste material do not compensate for this decrease in the cross-sectional area, although, for fraction III, where the waste material was as much as 30%, an improvement in the flexural strength was observed. In the case of compositions with an addition of 5% and 15%, the amount of dispersed reinforcement in the form of glass fibers is too small to have a real effect on the increase in flexural strength. The phenomenon observed for the composition with waste 1 consisting in a decrease in strength for the 15% additive (lower strength than for the 30% additive) is related to an increased porosity. This may be a result of improper mixing (aeration of the mixture). In photo 12, for the 15% addition of fraction I, significant pores are visible in large amounts (much more than for the 5% and 30% additions).

The geopolymer composite with waste fraction I was characterized by the worst strength properties and the highest porosity, which can be seen in Figure 12. Ultramacropores with diameter above 10 mm are very unfavorable. They have a very unfavorable influence on the strength properties. They are probably caused by the reaction of alkaline solutions with resin waste by the evolution of gases or by a significant aeration of the mixture during the manufacturing process. Although all samples were compacted, some reactions may have taken place in the material during curing, and it was not possible to completely remove these pores. It should be noted that this phenomenon is not accidental because, during numerous trials and repetitions, a very porous structure was obtained for the material with waste I each time.

The porosity of the test samples was determined by a comparative method through a visual analysis of the cross sections. No image analysis was performed because clear differences in porosity were observed on the cross sections, and the objective was only a qualitative rather than numerical comparative analysis.

The samples containing waste fraction I show the highest porosity among all of the tested geopolymer materials in the following work. It increases with an increase in the proportion of waste fraction. The cross sections of the samples with waste fraction II versus waste fraction III do not differ significantly. The colors of the different batches are significantly similar to each other, even though the waste fractions were different colors. The porosity of the samples with waste fraction III increases slightly with the proportion of the waste fraction amount. Pieces of fine river sand can be seen in the cross sections of the samples.

**Figure 15:**
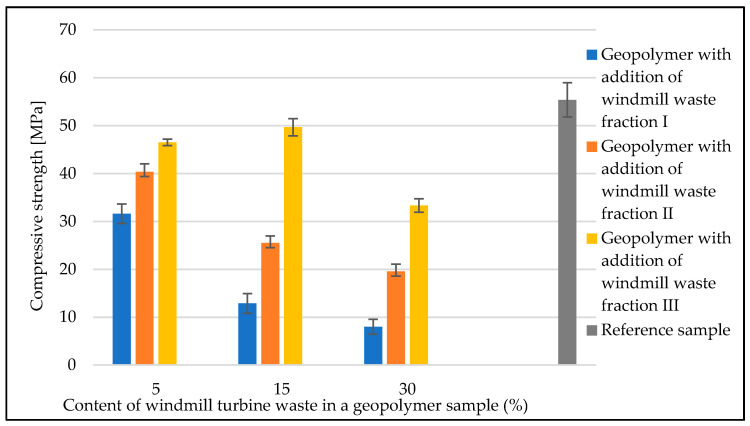
Compressive strength of geopolymer samples with the addition of windmill turbine waste fractions.

Figure 15 shows a summary of the average compression test results for the geopolymer samples based on fly ash and sand with the addition of the windmill turbine waste fraction. The average value for the reference sample was 55.4 MPa. The compressive strength of the materials with the addition of waste fractions was lower than that of the reference sample. The materials with waste fraction I and II showed a decrease in strength with an increasing filler amount. The material with waste fraction I in an amount of 5%, 15% and 30% showed the lowest strength in comparison to the other geopolymer materials, for which, the values were, respectively, 31.6 MPa (for 5% addition), 12.8 MPa (for 15% addition) and 8.0 MPa (for 30% addition).

The material with 5%, 15% and 30% waste fraction II showed an average compressive strength between waste fractions I and III. The average results obtained during this test were: 40.4 MPa (for 5% addition of fraction II), 25.5 MPa (for 15% addition of fraction II), and 19.6 MPa (for 30% addition of fraction II).

The materials with the addition of waste fraction III had the highest compressive strength. The addition of 15% fraction III to the geopolymer increased the compressive strength to 49.7 MPa. Samples with 5% and 30% filler showed a decrease in strength compared to the reference sample, and obtained an average strength of 46.5 MPa and 33.3 MPa, respectively.

### 3.5. Absorption Testing Results

**Figure 16:**
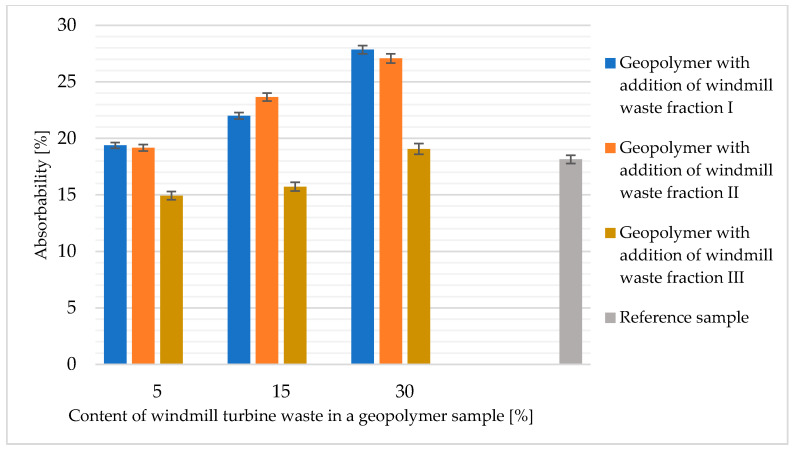
Absorbability of geopolymer samples based on fly ash and sand with the addition of waste from windmill turbines.

Figure 16 shows a summary of the results from the saturation test of fly-ash- and sand-based geopolymer samples with the addition of waste fractions from windmill turbines. The wettability increased for all samples with the amount of the waste phase filler contribution. For the samples with waste phase I and II in amounts of 5%, 15% and 30%, the absorbability values exceeded those obtained for the reference sample.

The material with 5% and 15% filling in the form of waste fraction III had a slightly lower absorbability than the reference sample. For the material with 30% waste fraction III, the absorbability value was slightly higher compared to the reference sample.

## 4. Conclusions

The studied composite materials made of waste from the rotor blades of wind power plants contained mainly glass fibers, where the analysis indicated the presence of elements such as Si, Ca, Al and O. This probably indicates the presence of R-type fibers, i.e., calcium–aluminum–silicate fibers. The glass fiber of this type also contains Mg and Na in its composition. These elements were also observed by EDS analysis, although the intensity of the peaks originating from these elements was low.

Potentially, the strength of the obtained materials could be higher if their porosity could be lowered. In this work, it has not been determined what exactly is the cause of the significant porosity in the materials. Factors such as the length of vibration, consistency of the mixture or molar concentration of the activator solution could affect the porosity parameter.

When considering waste disposal in a geopolymer material, it is important to keep in mind that such waste can arise from different parts of the wind turbine rotor blades. Additionally, different wind turbine models use different composite materials in varying proportions. Therefore, it seems that individually selected methods of producing the filler material should be applied to different milled waste fractions in order to obtain its optimal properties.

However, after analyzing the results, the filler in the form of ground waste fraction from wind turbine rotors shows a deterioration in the properties of the obtained material. It should be noted, however, that, for the waste fraction amounting to 5% and 15%, it is possible to obtain a material significantly different in absorbability, compressive strength and flexural strength than the material without the filler. The use of such a filler in the geopolymer matrix would potentially allow for both a significant utilization of waste material and lowering the price of geopolymer material production itself. During the LCA analysis, it should be kept in mind that this kind of waste material must be properly prepared in order to be used in concrete and geopolymer composites. At the moment, however, the waste is already processed in this way and properly ground, so the disposal of such processed waste should be considered as reducing the cost of geopolymers as a result of replacing ceramic fillers with the waste filler. Crushing/cutting/grinding should not be considered as an additional cost for geopolymers because these materials are processed in this way regardless of whether they are present in geopolymers. Of course, we emphasize again that any processing of this waste should be included in the LCA analysis.

## Figures and Tables

**Table 1:** List of geopolymers produced.

Index	Description	Mix Proportion (L/S)
K I 5	Geopolymer based on fraction I from windmill waste, river sand, fly ash (weight ratio: 0.05:1:1)	0.7:2.4
K I 15	Geopolymer based on fraction I from windmill waste, river sand, fly ash (weight ratio: 0.15:1:1)	0.55:2
K I 30	Geopolymer based on fraction I from windmill waste, river sand, fly ash (weight ratio: 0.3:1:1)	0.95:2.4
K II 5	Geopolymer based on fraction II from windmill waste, river sand, fly ash (weight ratio: 0.05:1:1)	0.6:2.2
K II 15	Geopolymer based on fraction II from windmill waste, river sand, fly ash (weight ratio: 0.15:1:1)	0.37: 1.1
K II 30	Geopolymer based on fraction II from windmill waste, river sand, fly ash (weight ratio: 0.3:1:1)	0.95:2.2
K III 5	Geopolymer based on fraction III from windmill waste, river sand, fly ash (weight ratio: 0.05:1:1)	0.53:2.4
K III 15	Geopolymer based on fraction III from windmill waste, river sand, fly ash (weight ratio: 0.15:1:1)	0.55:2.4
K III 30	Geopolymer based on fraction III from windmill waste, river sand, fly ash (weight ratio: 0.3:1:1)	0.72:2.4
R (reference sample)	Geopolymer based on river sand and fly ash (weight ratio: 1:1)	0.58:2

**Table 2:** Organic and inorganic content for specific waste fractions from wind turbines.

Fraction	Organic Content (%)	Inorganic Content (%)
I	27.65	72.35
II	43.11	56.89
III	29.13	70.87

## Data Availability

Not applicable.
